# Evaluation of Gait Performance of a Hemipelvectomy Amputation Walking with a Canadian Prosthesis

**DOI:** 10.1155/2014/962980

**Published:** 2014-04-14

**Authors:** M. T. Karimi, M. Kamali, H. Omar, Javid Mostmand

**Affiliations:** ^1^Faculty of Rehabilitation Sciences, Isfahan University of Medical Sciences, P.O. Box 81745-164, Isfahan, Iran; ^2^IUM University of Malaysia, Malaysia; ^3^Faculty of Rehabilitation Sciences, Isfahan University of Medical Sciences, Isfahan, Iran

## Abstract

*Background.* Hemipelvectomy amputation is a surgical procedure in which lower limb and a portion of pelvic are removed. There are a few studies in the literature regarding the performance of subjects with hip disarticulation during walking. However, there is no study on gait analysis of hemipelvectomy subject. Therefore, the aim of this paper was to evaluate the gait and stability of subject with hemipelvectomy amputation. *Case Description and Methods.* A subject with hemipelvectomy amputation at right side was involved in this study. He used a Canadian prosthesis with single axis ankle joint, 3R21 knee joint, and 7E7 hip joint for more than 10 years. The kinetic and kinematic parameters were collected by a motion analysis system and a Kistler force platform. *Findings and Outcomes.* There was a significant difference between knee, hip, and ankle range of motions and their moments in the sound and prosthesis sides. In the other side, the stability of the subject in the anteroposterior direction seems to be better than that in the mediolateral direction. *Conclusions.* There was a significant asymmetry between the kinetic and kinematic performance of the sound and prosthesis sides, which may be due to lack of muscular power and alignment of prosthesis components.

## 1. Introduction 

Lower limb amputations have been done as a result of trauma, vascular disease, cancer, and so forth. The incidence of amputation varies between 2.8 and 43.9 per 100000, in which 0.5% and 3% have been done as disarticulation through the hip joint or above [1, 2]. The main reasons for amputation at this level are vascular impairment, malignancy, and tumor [1, 3]. Those with hip disarticulation miss their abilities to stand and walk efficiently (without use of crutch or walker) and have to use especial prosthesis [2, 4]. Various types of components have been designed for hip disarticulation and hip pelvectomy to enable the subject to stand and walk [4, 5]; however, they have several problems including high energy consumption during walking, slow walking speed, style of walking which is not cosmetically appealing, and limitation in range of motion of leg joints [6–10]. As a result of small number of prosthetics users there is limited number of research on this topic. Furthermore, there are a few studies on kinetic and kinematic parameters of Canadian prosthesis users [6, 7, 9]. In one research study the loads applied on prosthesis were evaluated during walking of a subject with hip disarticulation amputation [8].

The mean walking speed of hip disarticulation (HD) subject (walking with prosthesis) varies between 0.83 and 1.31 m/s [7]. Moreover, their step length differed between 0.65 and 0.96 m, which is significantly less than that of normal subjects [7, 11]. The energy consumption of these amputees is also more than 2 times than that of normal subjects [11].

In contrast to hip disarticulation, hemipelvectomy amputation is a surgical procedure in which the lower limb is removed including a portion of the pelvic. Therefore, it seems that the performance of these subjects differs from those with HD, due to higher level of amputation. Based on author's knowledge, no research study evaluated the ability of subjects with amputation at this level. Therefore, the aim of this paper was to evaluate the performance of subject with this amputation.

## 2. Case Description and Methods

**Figure 1:**
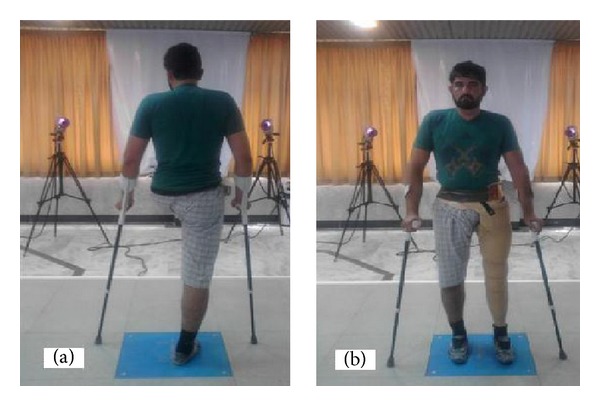
(a) Subject with hemipelvectomy amputation without prosthesis and (b) the subject while standing with a Canadian prosthesis.

**Figure 2:**
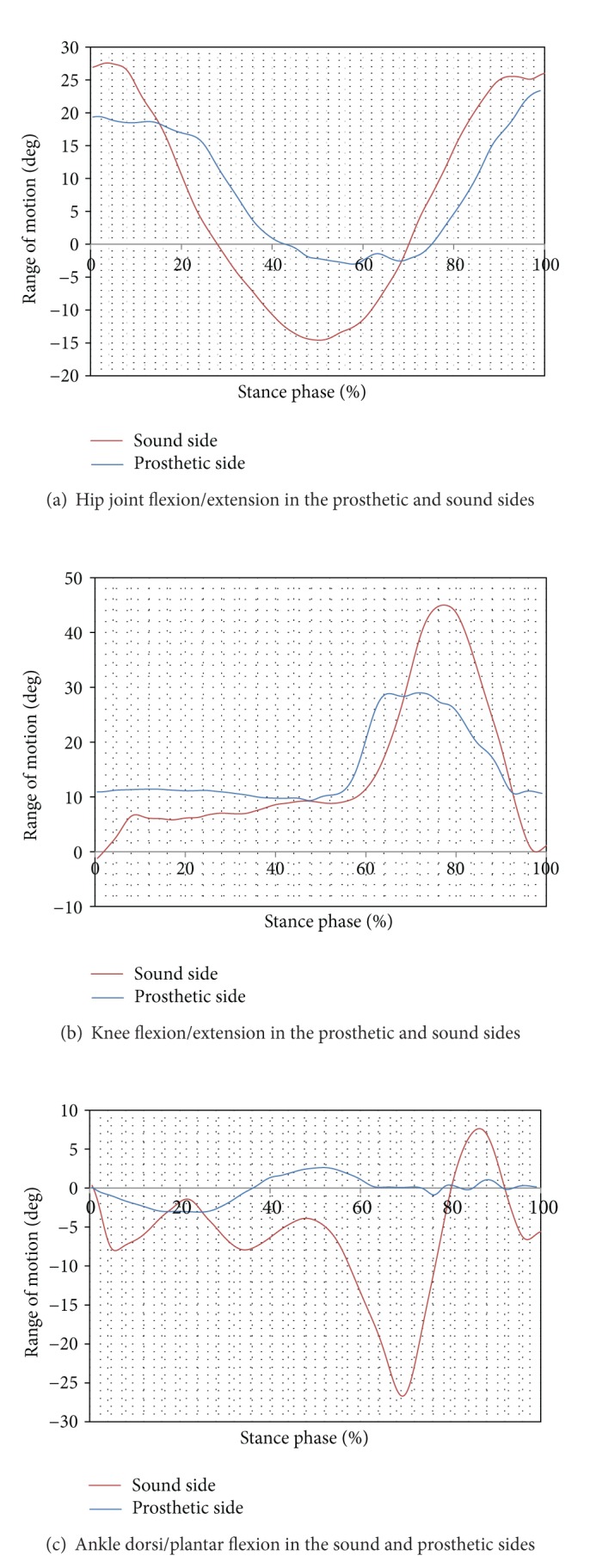


A subject with hemipelvectomy amputation at right side was involved in this study Figure 1(a). He used a Canadian prosthesis with single axis ankle joint, 3R21 knee joint, and 7E7 hip joint for more than 10 years Figure 2(b). An ethical approval was obtained from XXXXXX ethical committee. The subject was asked to sign a consent form before data collection. The mass, height, and age of the subject were 75 kg, 1.75 m, and 39 years, respectively.

### 2.1. Parameter

The spatiotemporal gait parameter (walking speed, cadence, stride length, and percentage of stance phase), the moments applied on the lower limb joints, the three planar motions of the lower limb joints and trunk, and the force applied on the legs during walking were the parameters collected in this study. Furthermore, the stability of the subject during standing was evaluated by use of a Kistler force plate when subject stands for one minute under eyes opened and closed conditions. The stability of the subject was evaluated by use of the following equations:

(1) $$\begin{array}{c} \text{COPEAP }\left(\text{mm}\right)=X_{\max}-X_{\min},\\ \text{COPEML}\;\left(\text{mm}\right)=Y_{\max}-Y_{\min},\\ \text{PLAP }\left(\text{mm}\right)=\overset{n-1}{\underset{}{\sum }}\sqrt{{\left(x_{i+1}-x_{i}\right)}^{2}},\\ \;\text{PLML }\left(\text{mm}\right)=\overset{n-1}{\underset{}{\sum }}\sqrt{{\left(y_{i+1}-y_{i}\right)}^{2}},\\ \text{VAP }\left(\text{mm}/\min\right)=\frac{\sum_{}^{n-1}\sqrt{{\left(x_{i+1}-x_{i}\right)}^{2}}}{t},\\ \text{VML }\left(\text{mm}/\min\right)=\frac{\sum_{}^{n-1}\sqrt{{\left(y_{i+1}-y_{i}\right)}^{2}}}{t}, \end{array}$$

where COPEAP, COPEML, PLAP, PLML, VAP, and VML are the excursion of the center of pressure in the anteroposterior direction, excursion of the center of pressure in the mediolateral direction, path length in the anteroposterior direction, path length in the mediolateral direction, velocity of the COP in the anteroposterior direction, and velocity of the COP in the mediolateral direction, respectively.

### 2.2. Procedure

Kinetic and kinematic assessments were performed in the gait lab using seven cameras of a three-dimensional (3D) gait analysis system (Qualisys Motion Analysis System) and a force plate (Kistler). The data were analyzed using visual 3D software, developed by C-Motion, Inc., which allows calculation of the forces and moments of different joints from the collected raw data. The frequency of force plate and cameras was set at 120 Hz. The participant was asked to walk and stand along the gait lab. The markers used in this research were delete of spherical type with a diameter of 14 mm covered with a reflective sheet that could be detected by the cameras. The markers were attached to the legs and pelvis according to the preferred method of marker adhesion and subsequent identification used in the Bioengineering Unit of the University of Strathclyde. In total, 16 markers were attached to the right and left anterior superior iliac spine (ASIS), right and left posterior superior iliac spine (PSIS), medial and lateral malleolus, first and fifth metatarsal heads, and right and left greater trochanter (R-L GT). Moreover, four-markers cluster comprising four markers attached to rhomboid plates were attached to the anterior surface of the legs and thighs using extensible Velcro straps. The participant was asked to walk with a comfortable speed along the gait lab (it should be mentioned that the subject used crutches during walking). The tests were repeated 10 times while subject using a Canadian prosthesis. The two samples *t*-test with a significant point at 0.05 was used for final analysis. The statistical test was done using SPSS software (version 21).

## 3. Findings and Outcomes

The range of motion of the ankle, knee and hip joints in three planes is shown in Table 1. As can  be seen from this table the range of motion of ankle joint in sagittal, coronal, and transverse planes was 34.18 ± 2.12, 17.45 ± 1.28 and 13.92 ± 2.14 degrees, in the sound side, respectively, compared to 4.96 ± 0.12, 2.95 ± 0.19, and 6.88 ± 1.02 in the prosthesis side, respectively. There was a significant difference between knee range of motion in the sound and prosthesis sides (*P*  value < 0.05). The range of motion of hip joint was 40.3 in the sound side compared to 27.33 in prosthesis side (*P*  value = 0.001). The hip joint abduction/adduction excursion was 7.61 in normal side compared to 6.9 in the amputed side (*P*  value > 0.05).

Table 2 summarizes the kinematic parameters of pelvic and thorax in normal and amputed sides. As can be seen from this table there was no difference between range of motions of the pelvic in right and left sides in sagittal and transverse planes. The mean values of forward and lateral bending of trunk were 14.03 ± 2.01 and 2.59 ± 0.06 degrees in normal side and 11.06 ± 2.02 and 3.22 ± 1 in the prosthesis side, respectively (*P*  value < 0.05). The moment applied on the hip joint was the other parameter selected in this research study. The mean value of flexion moment of the hip joint was 0.32 ± 0.001 and 0.187 ± 0.01 Nm/body mass in the sound and prosthetic sides, respectively (*P*  value = 0.01). The extension moment of the hip joint in normal side was two times more than that in prosthetic side. Although, there was a significant difference between adductor moment of the hip joint in both sides, the difference between the mean values of the moments transmitted through knee joint was not significant. As can be seen from Table 3, the moments of the knee joint in prosthetic side differed significantly from those of the sound side. Figures 2(a), 2(b), and 2(c) show the motions of the hip, knee, and ankle joints in the normal and prosthetic sides.

Table 3 shows the mean values of the moments applied on the ankle joint and the force transmitted through both sides. The mean value of vertical force applied on the left leg was 865.8 ± 56.22 N compared to 245.8 ± 22.22 N on the right side (*P*  value = 0). The first and second peaks of anteroposterior force were 51 ± 6.12 and 110 ± 17.12 N in the normal side compared to 9.16 ± 1.25 and 15.31 ± 1.23 in the prosthesis side (*P*  value = 0). The moment applied on the ankle joint in the amputed side is significantly less than that of normal side.

## 4. Discussion

Hemipelvectomy amputation is a surgical procedure in which the lower limb and a portion of pelvic are removed. There were a few studies in the literature regarding the performance of subjects with hip disarticulation during walking. However, there was no study on gait analysis of hemipelvectomy subject. Therefore, the aim of this case study was to evaluate the gait and stability of subject with hemipelvectomy.

As can be seen from Table 1, the kinematic of the hip joint, knee, and ankle joints differed significantly between normal and amputee sides. The range of motion of the hip joint in sagittal plane was 40.3 in normal side compared to 27.33 in prosthesis side. The main reason was related to the position of the hip joint in prosthesis side which is located on the anterior side of the socket [5]. The range of knee joint flexion/extension was also less than normal side, which may be due to the alignment of the prosthesis components [2, 5]. The main strategy used by amputees to control the motion of the artificial joints is to change the location of center of gravity (COG) with respect to the center of the joint. The alignment of this prosthesis components is done in which the load line always passes in front of knee joint and behind the hip joint; therefore, the hip and knee joints are always kept in extension during stance phase [2, 7].

Regarding the motion of pelvic, there was no significant difference between flexion/extension and rotation of pelvic in both sides. The main reason was related to the especial design of socket of this prosthesis which encloses the pelvic [4, 7]. Therefore, the range of motion of pelvic was significantly restricted in both sides as the upper brim of the socket also surrounded the trunk.

The other parameter which has been used in this research study was the moment applied on the hip joint. The moment of the hip joint in the prosthesis side was significantly less than that in normal side. The main reason may be related to the force transmitted through the leg in the prosthesis side. The mean values of flexion, extension, and abduction moments of the hip joint in normal subjects are 0.4 to 0.98, 0.75 to 0.98, and 0.97 Nm/BM, respectively [11], which is nearly the same as the moment applied on the sound side, in this research. Restricted range of motion in prosthesis side may be the other reason for this difference. In the research done by Nietert et al. the flexion and abduction moments of the hip joint were 1.1 and 0.98 Nm/BM, respectively [8], which are more than the results of the current research. The main reason for this difference may be related to use of crutch in this research. The subject used crutch to improve his balance during walking and standing. Some portion of body weight was applied on the crutch which influenced the moments transmitted through the prosthesis component.

The forces transmitted through both legs differed significantly. In both sides the magnitude of breaking and progression forces varied significantly. However, in normal subject these forces are nearly the same, which represent the symmetry of walking [11]. However in prosthesis side, not only the mean value decreased significantly, but also the values of the first and second peaks were not the same. The main reasons may be related to lack of muscular power in the prosthesis side and also use of the crutch.

The mean values of stability parameters of the participant under eyes opened and closed conditions are shown in Table 4. The excursions of COP in the mediolateral and anteroposterior directions in open eyes condition were 5.056 ± 1.87 and 4.22 ± 0.54 mm, respectively. The velocity of COP sway in the anteroposterior direction was 885 ± 135.9 mm, compared to 1020.62 ± 627 mm in the mediolateral direction. Based on this parameter, the stability of the subject in the anteroposterior direction seems to be better than that in the mediolateral direction.

Unfortunately, there is no study in the literature regarding the stability of subject with hemipelvectomy amputation. This case study is the first study done in this regard. As can be seen from Table 4, the mean value of excursion of COP was 4.22 mm in the anteroposterior and 5.05 mm in the mediolateral direction. The mean value of COP excursion of normal subject in the anteroposterior and mediolateral directions was 29 ± 4 and 14 ± 4 mm, respectively [12]. It seems that the subject was more stable than normal subjects. However, it should be emphasized that in this study the stability was evaluated during quiet standing. The results of stability of quiet standing also represented the suitability of crutch to improve standing stability. It should be emphasized that as there was no muscular support around the hip, knee, and ankle joints the subject was not able to have a dynamic stability. Moreover, his behavior may differ from normal subject when a protrusion force was applied. The alignment of prosthesis components may be the other reason for better stability of the subject. The alignment of prosthesis component keeps the leg in an extended posture which is quite stable during standing [7].

## 5. Conclusion

Although there are few studies on the gait analysis of subject with hip disarticulation amputation, there is no study on hemipelvectomy amputation. The result of this research showed that there was a significant asymmetry between the kinetic and kinematic performance of the sound and prosthesis sides which may be due to lack of muscular power and alignment of prosthesis component. It has been recommended to use the data of this study to design prosthesis components.

## Figures and Tables

**Table 1:** The range of motion of the ankle, knee, and hip joints in three planes.

Parameters	Ankle flexion/extension	Ankle inversion/eversion	Ankle rotation	Knee flexion/extension	Knee abduction/adduction	Knee rotation	Hip flexion/extension	Hip abduction/adduction	Hip rotation
Sound side	34.18 ± 2.12	17.45 ± 1.28	13.92 ± 2.14	46.95 ± 6.52	15 ± 3.25	19.71 ± 2.12	40.3 ± 7.16	7.61 ± 1.20	9.46 ± 1.12
Prosthesis side	4.96 ± 0.12	2.95 ± 0.19	6.88 ± 1.02	24.98 ± 3.85	7.66 ± 1.28	4.59 ± 0.15	27.33 ± 3.65	6.9 ± 0.92	3.5 ± 1.2
*P* value	0	0.002	0.004	0	0.01	0	0.001	0.3	0.32

**Table 2:** The kinematic parameters of pelvic and thorax in normal and amputed sides.

Parameter	Pelvic flexion/extension	Pelvic abduction/adduction	Pelvic rotation	Thorax flexion/extension	Thorax abduction/adduction	Thorax rotation	Hip moment (flexion)	Hip moment (extension)	Hip moment (adduction)	Hip moment (external rotation)	Hip moment (internal rotation)
Sound side	3.34 ± 0.15	18.05 ± 0.08	6.37 ± 1.05	14.03 ± 2.01	2.59 ± 0.06	4.9 ± 0.02	0.32 ± 0.001	0.8114 ± 0.06	1.21 ± 0.01	0.039 ± 0.0006	0.075 ± 0.002
Prosthesis side	2.89 ± 0.01	14.318 ± 1.02	5.61 ± 1.00	11.06 ± 2.02	3.22 ± 1.00	4.84 ± 0.99	0.187 ± 0.01	0.31 ± 0.004	0.358 ± 0.007	0.034 ± 0.002	0.046 ± 0.02
*P*-value	0.1	0	0.24	0.035	0.017	0.39	0.01	0	0	0.33	0.056

**Table 3:** The mean values of the moment applied on the ankle and knee joints and the force transmitted through both sides.

Parameter	Knee moment extension	Ankle moment plantar flexion	Ankle moment inversion/eversion	Ankle moment rotation	FX1	FX2	FY	FZ	Knee moment flexion	Knee moment adduction	Knee moment rotation
Sound side	0.147 ± 0.07	1.432 ± 0.002	0.528 ± 0.003	0.242 ± 0.01	51 ± 6.12	110 ± 17.12	27 ± 9.23	865.8 ± 56.22	0.44 ± 0.15	0.32 ± 0.0012	0.09 ± 0.01
Prosthesis side	0.0202 ± 0.003	0.37 ± 0.01	0.116 ± 0.012	0.12 ± 0.02	9.16 ± 1.25	15.31 ± 1.23	19.66 ± 1.28	248.8 ± 22.22	0.126 ± 0.12	0.1 ± 0.0007	0.05 ± 00.1
*P*-value	0	0	0	0	0	0	0	0	0.003	0.008	0.001

**Table 4:** The mean value of stability parameters of the participant under eyes opened and closed condition.

Parameter	Mean value	
	Open eye	Closed eye
Path length *x*	425.506 ± 67.97	412.216 ± 37.149
Path length *y*	510.313 ± 47.93	403.636 ± 5.37
Cop *x* excursion	5.056 ± 1.874	3.19 ± 0.54
Cop *y* excursion	4.227 ± 0.54	1.568 ± 0.447
Velocity cop *x*	885.013 ± 135.95	824.433 ± 74.298
Velocity cop *y*	1020.627 ± 149.86	807.273 ± 10.74
